# The role of premium subsidies in crop insurance

**DOI:** 10.1371/journal.pone.0250129

**Published:** 2021-04-13

**Authors:** Charalampos Mavroutsikos, Konstantinos Giannakas, Cory Walters

**Affiliations:** Department of Agricultural Economics, University of Nebraska-Lincoln, Lincoln, Nebraska, United States of America; Szechenyi Istvan University, HUNGARY

## Abstract

This study develops a novel framework of heterogeneous producer attitudes towards risk to analyze different, stated and revealed, roles of crop insurance premium subsidies and underlying policy objectives of the government. The analysis reveals a strong connection and a complementarity between the roles of premium subsidies in increasing producer participation in crop insurance, inducing a desired separating equilibrium in the presence of asymmetric information, and transferring income to agricultural producers participating in the program. Developing an alternative design of premium subsidies that can achieve the stated government objective of increased producer participation and induce any desired separating equilibrium at significantly reduced costs, our study rejects the idea that the income redistribution taking place under the current policy design is necessary for increasing producer participation in crop insurance. Indeed, the current policy design reveals that premium subsidies are either a means of income redistribution or a policy failure.

## Introduction

The U.S. federal crop insurance is a major farm policy aimed at providing risk protection/reduced risk exposure to agricultural producers [1, 2]. A key component of this policy is the provision of multiple contract options and subsidies that reduce the cost of insurance to agricultural producers [3–6]. Premium subsidies accounted for $6.26 billion in government outlays in 2019, with $2 billion being applied to coverage levels of 80% and higher [7]. While the government has justified the use of premium subsidies as a necessary means of increasing producer participation in crop insurance [4, 8–14], many have argued that premium subsidies are just another means of income redistribution from taxpayers to producers [5, 6, 15–17]. As pointed out by an anonymous reviewer and many of the cited studies, key reasons behind the government objective of increased producer participation in crop insurance have been a desire to reduce adverse selection, increase the accuracy of premium rates, and eliminate ad-hoc disaster payment programs.

Given the significant producer heterogeneity with respect to attitudes towards risk [18–21] and the fact that these attitudes are private information, an argument can also be made that premium subsidies are a means of resolving this information asymmetry and inducing certain insurance contract choices by producers. Indeed, the provision of multiple insurance contracts reveals the government’s objective of inducing a separating equilibrium (where producers select from a menu of contracts based on their risk preferences) and the premium subsidies represent a necessary means of achieving this objective.

The objective of this study is to analyze and evaluate all different policy objectives/roles of premium subsidies and improve our understanding of the relationship between the stated and revealed government objectives and the role of premium subsidies in achieving these objectives.

To study the role of premium subsidies in crop insurance policy design and implementation, our study develops a novel framework of analysis that explicitly accounts for the empirically relevant heterogeneity in producer attitudes towards risk and the multiple insurance options/contracts available to them. The explicit consideration of heterogeneous producer risk preferences is important in understanding the partial producer participation in crop insurance, differences in producer choices regarding insurance coverage as well as the asymmetric effects of the policy on producers with different risk preferences.

The analysis reveals a strong connection and a complementarity between the stated and revealed policy objectives of the government. Using insights from our model, we identify an alternative design of premium subsidies that can achieve the stated government objective of increased producer participation and induce any desired separating equilibrium at reduced costs. The existence of such a mechanism rejects the idea that the income redistribution taking place under the current policy design is necessary for increasing producer participation in crop insurance.

Overall, our manuscript makes three contributions to the literature. First, it introduces a novel framework of analysis that effectively captures the empirically relevant heterogeneity in producer attitudes towards risk (which has been ignored by the relevant literature) as well as the tradeoffs involved in producer insurance decisions in a parsimonious manner. Second, it develops and proposes an alternative design of the crop insurance policy mechanism that can increase producer participation (which is the stated goal of the program) and induce any separating equilibrium at significantly lower costs. Third, by developing this new policy design, the analysis reveals that the premium subsidies in the current policy design are either a means of income redistribution or a policy failure.

The rest of this manuscript is organized as follows. The next section provides some background information on the importance of premium subsidies in the evolution of crop insurance. Next, we introduce the theoretical framework of heterogeneous producer attitudes towards risk and analyze the producer decisions and welfare under the crop insurance policy. We then discuss the different roles of the premium subsidies in crop insurance, and present a policy design that can achieve increased producer participation and any desired separating equilibrium at reduced taxpayer costs. The final section summarizes and concludes the study.

### Background information on crop insurance and premium subsidies

The inception of the Federal Crop Insurance Program dates back to 1938, when it was initiated as a risk protection mechanism following the dust bowl and unsuccessful attempts by the private industry to provide insurance to agricultural producers [22]. Crop insurance as part of the U.S. Federal Government farm program started as a pilot program with limited crop and regional insurance coverage, low producer participation and high loss experience. After a phase of program experimentation and amendments intended to improve a costly program with low producer participation, the Federal Crop Insurance Act of 1980 introduced premium subsidies as well as new forms of policy coverage and insured crops [23].

Subsequent legislative reforms focusing on the structure and magnitude of premium subsidies further increased the size and scope of the program that ended up providing nationwide multi-peril crop insurance coverage for almost all available crops and commodities. Most notable among those reforms are the Federal Crop Insurance Reform Act of 1994 that increased premium subsidies to enhance producer participation in the program [23, 24], the 1996 Farm Act that approved the development and provision of revenue-based policies [14, 24], and the 2000 Agricultural Risk Protection Act (ARPA) that provided $8.2 billion for the development of new insurance products and additional premium subsidies [15]. ARPA changed also the structure of premium subsidies for revenue-based policies from fixed per acre dollar amounts to percentage shares of the premiums, the same subsidy structure found in yield-based policies [25]. The subsequent increase in producer participation rates in new insurance products covering new crops and new areas, and the selection of higher insurance coverage options have resulted in a program that grew from $200 million in premium subsidies in 1989 to $6.26 billion in premium subsidies in 2019 [4, 5, 7].

### Theoretical framework–producer characteristics and behavior

Due to differences in their attitudes towards risk and level of risk aversion, agricultural producers differ in the costs they incur for being involved in a risky endeavor like agricultural production. The greater a producer’s aversion to risk, the greater the perceived costs of operating/producing under a given level of risk exposure, and the lower the expected net returns to production [26]. To reduce their exposure to risk (and, through this, their costs of operating in a risky environment), producers can purchase a crop insurance contract at the beginning of the production year. The greater the risk protection/reduction in risk exposure provided by a crop insurance contract, the greater the premium associated with the procurement of the contract.

To capture these elements, consider a continuum of agricultural producers differing in the level of their aversion to risk that have the choice between self-insuring, purchasing a low-coverage insurance policy, and purchasing a high-coverage policy. Note that, in reality, there are multiple insurance policies with different levels of risk coverage available to producers. While our model can be easily adapted to consider a large number of crop insurance contracts, such consideration would complicate the analysis without affecting the qualitative nature of our results.

Let *A* ∈ [0.1] be the attribute that differentiates producers with greater values of *A* corresponding to greater aversion to risk. The expected net returns associated with the different options for the producer with differentiating attribute *A* are given by:

$$\begin{array}{l} ER_{si}=ERP-\beta A\;\text{under}\;\text{self-insurance}\\ {ER}_{l}=ERP-w_{l}-\gamma A\;\text{under}\;\text{low-coverage}\;\text{crop}\;\text{insurance}\\ ER_{h}=ERP-w_{h}-\delta A\;\text{under}\;\text{high-coverage}\;\text{crop}\;\text{insurance} \end{array}$$ (1)

where *ER*_*si*_, *ER*_*l*_ and *ER*_*h*_ are the expected net returns from agricultural production under self-insurance, low-coverage crop insurance and high-coverage crop insurance, respectively; *ERP* are the expected returns to agricultural production that are common across producers; *w*_*l*_ and *w*_*h*_ are the costs of low-coverage and high-coverage crop insurance, respectively, with *w*_*h*_ > *w*_*l*_ and *β*, *γ* and *δ* are risk exposure parameters capturing the producer risk exposure under self-insurance, low-coverage crop insurance and high-coverage crop insurance, respectively. The *ERP* are given by (*p-c*)*q* where *p* is the farm price of the agricultural product, *c* is the cost of producing this product, and *q* is the quantity produced. Depending on the source of uncertainty (i.e., whether there is price, cost or yield uncertainty), the parameters *p*, *c* and *q* would represent the expected price, expected cost and expected output, respectively. As pointed out by an anonymous reviewer, the expected returns to production could vary among the different insurance options if, due to moral hazard, the procurement of insurance affected producers’ effort and production decisions. While consideration of differences in the expected returns to production would complicate the analysis, it would not affect the results of our study.

To capture the reduction in risk exposure due to insurance coverage, we assume that *β* > *γ* > *δ*. In this context, *βA*, *γA* and *δA* represent the costs associated with (risky) agricultural production under the different insurance options for the producer with differentiating attribute *A*. The greater the riskiness of agricultural production/producer exposure to risk and/or the greater the producer aversion to risk, the greater the producer costs of being involved in this risky endeavor.

**Fig 1:**
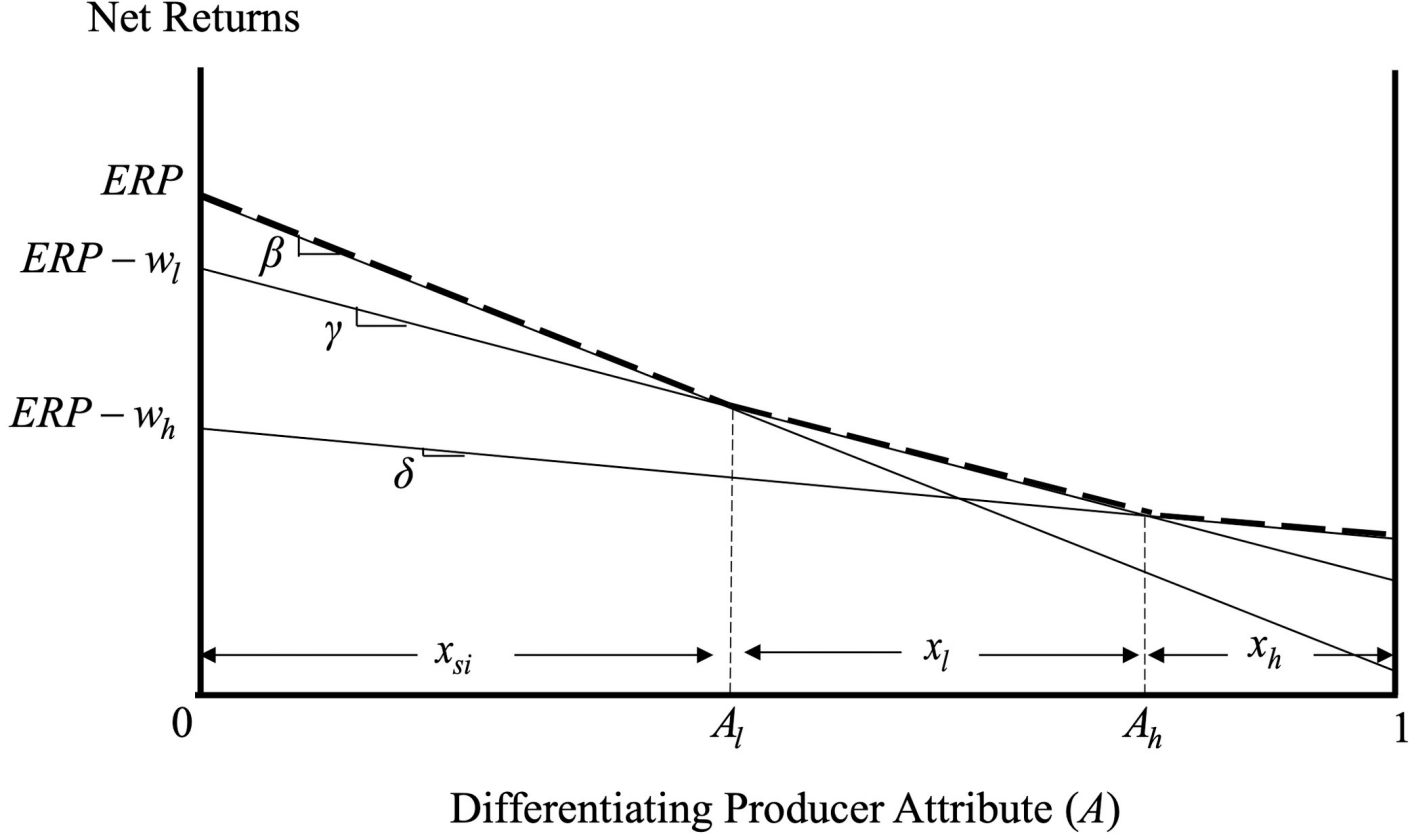
Producer insurance decisions and welfare.

The producer insurance decision depends on the relative expected net returns associated with self-insurance, low-coverage crop insurance and high-coverage crop insurance. Fig 1 graphs *ER*_*si*_, *ER*_*l*_ and *ER*_*h*_ for the empirically relevant case where the different insurance options enjoy positive shares of the market.

The producer with risk aversion

$$A_{l}:ER_{si}=ER_{l}\Rightarrow A_{l}=\frac{w_{l}}{\beta -\gamma }$$ (2)

is indifferent between self-insurance and low-coverage crop insurance as the expected net returns associated with the two options are the same. Similarly, the producer with differentiating attribute

$$A_{h}:ER_{l}=ER_{h}\Rightarrow A_{h}=\frac{w_{h}-w_{l}}{\gamma -\delta }$$ (3)

is indifferent between the low-coverage and the high-coverage insurance options.

Producers with relatively low risk aversion (i.e., producers with *A* ∈[0.*A*_*l*_)) prefer to self-insure, producers with intermediate levels of risk aversion *A* ∈ (*A*_*l*_, *A*_*h*_) prefer the low-coverage crop-insurance, while more risk averse producers with *A* ∈ (*A*_*h*_,1] prefer the high-coverage crop insurance. Assuming, for simplicity and without loss of generality, that producers are uniformly distributed with respect to their aversion to risk, *A*_*l*_ gives the share of producers that prefer to self-insure, *x*_*si*_;*A*_*h*_—*A*_*l*_ is the share of producers that buy low-coverge crop insurance, *x*_*l*_; and 1 − *A*_*h*_ is the share of producers that prefer the high-coverage crop insurance, *x*_*h*_. Mathematically, *x*_*si*_, *x*_*l*_ and *x*_*h*_ are given by:

$$x_{si}=\frac{w_{l}}{\beta -\gamma }$$ (4)

$$x_{l}=\frac{w_{h}(\beta -\gamma )-w_{l}(\beta -\delta )}{\left(\beta -\gamma )(\gamma -\delta \right)}$$ (5)

$$x_{h}=\frac{\left(\gamma -\delta )-(w_{h}-w_{l}\right)}{\gamma -\delta }$$ (6)

The greater the reduction in risk exposure and/or the lower the costs associated with the low-coverage crop insurance, the greater the share of producers that find it optimal to participate in crop insurance. Similarly, the greater the reduction in risk exposure and/or the lower the costs associated with a particular crop insurance contract, the greater the share of producers that prefer this crop insurance contract.

Before concluding this section, it is important to note that, in the spirit of [27], our simple model captures the essence of risk aversion and its impact, along with risk exposure, on crop insurance decisions without adhering to expected utility theory and its calibration/scaling limitations [28, 29]. This is particularly important in our case as agricultural producers can enrol from a few hundrend to thousands of acres in crop insurance. In addition to depicting the insurance decisions of heterogeneous agricultural producers, the area under the effective (dashed and kinked) expected net returns curve in Fig 1 graphs also the welfare of different producers under the different insurance options. Specifically, the welfare of producers that self-insure, producers that buy low-coverage crop insurance, and producers that buy high-coverage crop insurance (denoted by *PW*_*si*_, *PW*_*l*_ and *PW*_*h*_, respectively) are given by:

$$PW_{si}=\int_{0}^{A_{l}}ER_{si}\;dA$$ (7)

$$PW_{l}=\int_{A_{l}}^{A_{h}}ER_{l}\;dA$$ (8)

$$PW_{h}=\int_{A_{h}}^{1}ER_{h}\;dA$$ (9)

These welfare measures will become important when we discuss the distributional impacts of premium subsidies.

### The role of premium subsidies in crop insurance

As noted earlier, an important component of the crop insurance policy are the premium subsidies for the different crop insurance contract options. These premium subsidies are designed to reduce the insurance costs to producers and, in their presence, the producer expected net returns function becomes:

$$\begin{array}{l} ER_{si}=ERP-\beta A\;\text{under}\;\text{self-insurance}\\ {ER}_{ls}=ERP-w_{l}(1-s_{l})-\gamma A\;\text{under}\;\text{low-coverage}\;\text{crop}\;\text{insurance}\\ ER_{hs}=ERP-w_{h}(1-s_{h})-\delta A\;\text{under}\;\text{high-coverage}\;\text{crop}\;\text{insurance} \end{array}$$ (10)

where *s*_*l*_ and *s*_*h*_ are the government determined premium subsidies for low- and high-coverage crop insurance, respectively. These subsidies are proportional to the insurance costs/premiums *w*_*l*_ and *w*_*h*_ taking, therefore, values between 0 and 1, and decline as coverage levels increase (i.e., *s*_*l*_ > *s*_*h*_). The greater is the value of a premium subsidy, the lower the costs of the specific crop insurance coverage contract, and the greater the expected net returns associated with this insurance contract, i.e., $\frac{\partial ER_{ls}}{\partial s_{l}}>0$ and $\frac{\partial ER_{hs}}{\partial s_{h}}>0$.

**Fig 2:**
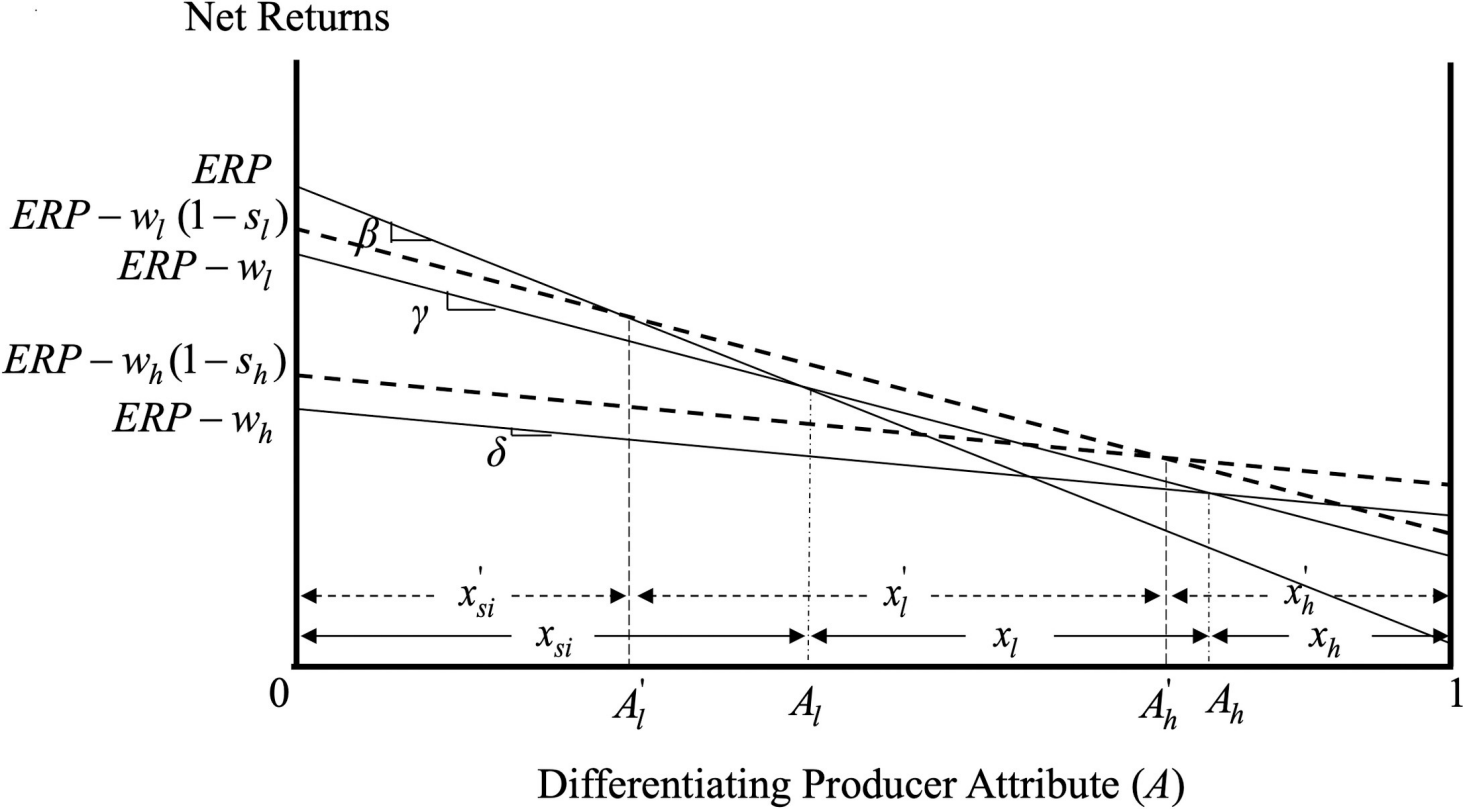
Market and welfare effects of premium subsidies when *s*_*l*_*w*_*l*_ < *s*_*h*_*w*_*h*_.

All other variables are as defined previously. Fig 2 graphs the impact of premium subsidies on the producer expected net returns associated with the different insurance options with the dashed curves depicting the expected returns associated with the low- and high-coverage crop insurance in the presence of the subsidies. While the study focuses on the introduction of new subsidies, the analysis is more general and applies also to cases where the government increases the magnitude of its existing subsidies.

### Premium subsidies as means of increasing producer participation in crop insurance

As shown in Fig 2, by reducing the insurance costs to producers, the introduction of the premium subsidies causes an upward parallel shift of the expected net returns curves associated with the crop insurance options. Regarding the increased producer participation in crop insurance, which, as noted earlier, is a key stated government objective of the utilization of premium subsidies, our model shows that subsidizing the low-coverage crop insurance does result in increased producer participation in the program. Specifically, the increased expected net returns in the presence of the subsidies result in increased producer participation in the program by $A_{l}-A_{l}^{'}$ in Fig 2 as producers with differentiating attribute/level of risk aversion $A\in (A_{l}^{'},A_{l}]$ that were initially self-insuring find it optimal to purchase low-coverage crop insurance in the presence of the premium subsidies. Mathematically, the increase in the producer participation in the presence of the premium subsidies is given by

$$A_{l}-A_{l}^{'}=\frac{w_{l}s_{l}}{\beta -\gamma }$$ (11)

The greater the premium subsidy for low-coverage crop insurance, the greater the share of producers that find it optimal to participate in crop insurance.

**Result 1**: The introduction of crop insurance premium subsidies increases producer participation in the policy, which is the stated goal of the government.

Crop insurance data from the Risk Management Agency (RMA), available in S1 Data, is consistent with the positive impact of premium subsidies on producer participation in crop insurance over time [7]. Through the implementation of several legislative acts expanding premium subsidies, the crop insurance program grew from $254.8 million in premium subsidies for 99.6 million insured acres in 1994 to $6.26 billion in premium subsidies for 769 million insured acres in 2019.

### Premium subsidies as means of inducing a desirable separating equilibrium in the presence of asymmetric information

The provision of multiple crop insurance contract options to agricultural producers reveals the government’s desire to have different producers/producers with different attitudes towards risk choose different insurance coverage contracts. Obviously, if this were not the case, i.e., if the government wanted all producers to choose the same insurance coverage, it could/would have, simply, made one crop insurance coverage option available to producers. Put in a different way, the provision of multiple insurance coverage contracts reveals the government’s desire for the existence of a separating equilibrium where producers with different levels of risk aversion choose different insurance contracts.

With the producer attitudes towards risk/level of risk aversion being private information, this represents a typical case of asymmetric information where the type of the agent/informed party is hidden to the principal/uniformed party. In such a case, the provision of a menu of contracts is part of the appropriate response of the uninformed party (government in our case) that desires to resolve the information asymmetry and have different agents/producers choose the contract designed for them. Subsidies linked to the different contracts (like the premium subsidies for low- and high-coverage crop insurance policies, in our case) can then be used to satisfy incentive compatibility and induce different producer groups choose the policies designed for them [30].

In the context of our study, the provision of low- and high-coverage insurance policies reveals the government’s desire to have producers with intermediate and high aversion to risk choose low- and high-coverage crop insurance contract, respectively, with the premium subsidies helping determine the desirable threshold levels of risk aversion (i.e., the levels of risk aversion that determine the desirable boundaries of “intermediate” and “high” risk aversion) and the share of producers under each insurance contract.

In the context of the analysis in the previous section, for instance, in addition to increasing producer participation in crop insurance, subsidizing the low-coverage insurance policy also changes the share of producers that purchase a high-coverage crop insurance contract as a number of them find it optimal to switch to the low-coverage policy in the presence of *s*_*l*_. If the government is interested in maintaining/achieving a certain share of producers in the high-coverage option, then it has to also subsidize the high-coverage policy. Note that, twenty years after the introduction of 80% and 85% coverage levels in 1999, high-coverage policies grew to represent 26% of insured acres and 32% of premium subsidies.

To further illustrate this role of premium subsidies, assume that the current levels of producer participation in low- and high-coverage crop insurance are given by *x*_*l*_ and *x*_*h*_ in Fig 2, respectively, and that the government wishes to increase them to $x_{l}^{'}$ and $x_{h}^{'}$ in the same Figure. This is equivalent to saying that the government desires producers with risk aversion in excess of $A_{h}^{'}$ to purchase high-coverage insurance and producers with risk aversion $A\in (A_{l}^{'},A_{h}^{'}]$ to purchase low-coverage crop insurance. Carefully designing *s*_*l*_ and *s*_*h*_, the government can achieve this (and any other) change in the shares (and, thus, types) of producers choosing the different insurance coverage contracts, as those shares are, indeed, functions of the relative subsidies received by the two contracts, i.e.,

$$x_{l}^{'}=\frac{w_{h}(\beta -\gamma )-w_{l}(\beta -\delta )-w_{h}s_{h}(\beta -\gamma )+w_{l}s_{l}(\beta -\delta )}{\left(\beta -\gamma )(\gamma -\delta \right)}$$ (12)

$$x_{h}^{'}=\frac{\left(\gamma -\delta )-(w_{h}-w_{l})+(w_{h}s_{h}-w_{l}s_{l}\right)}{\gamma -\delta }$$ (13)

**Fig 3:**
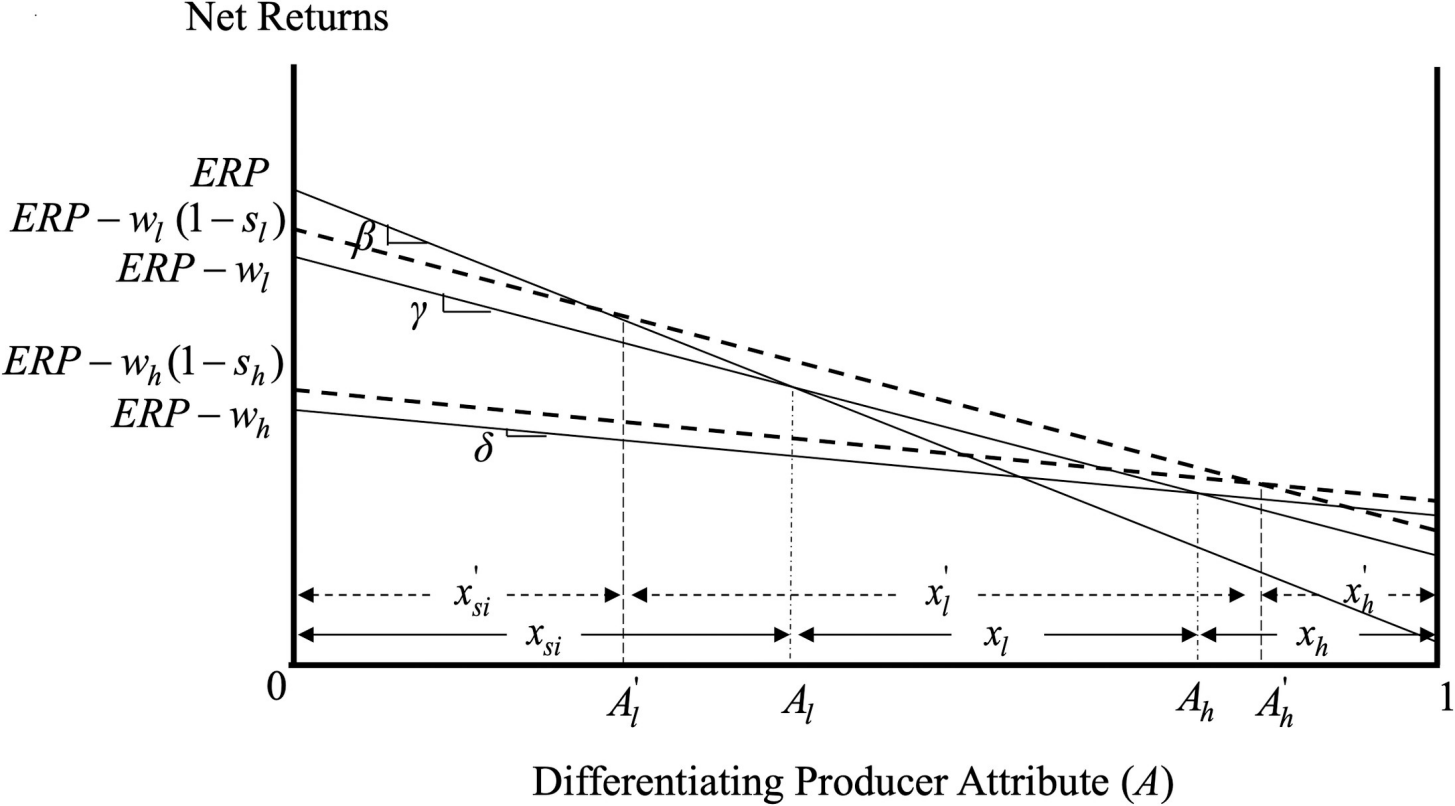
Market and welfare effects of premium subsidies when *s*_*l*_*w*_*l*_ < *s*_*h*_*w*_*h*_.

Note that, while Fig 2 depicts the case where *s*_*l*_*w*_*l*_ is less than *s*_*h*_*w*_*h*_ inducing an increased number of producers to opt for the high-coverage crop insurance contract, the premium subsidies can be structured such that the share of producers opting for the high-coverage insurance contract is reduced or stays the same. For instance, while ARPA resulted in a regressive proportional scheme of the premium subsidy rates (i.e., *s*_*h*_<*s*_*l*_), the dollar amount of the premium subsidy can still increase with the coverage level (i.e., *s*_*h*_*w*_*h*_ > *s*_*l*_*w*_*l*_), as the difference in the subsidy rates can be outweighed by the difference in the insurance premiums *w*_*h*_ and *w*_*l*_ [25]. Fig 3 depicts the case where *s*_*l*_*w*_*l*_ exceeds *s*_*h*_*w*_*h*_ and producers who initially had a high-coverage insurance contract (producers with $A\in (A_{h},A_{h}^{'}]$ in this Figure) find it optimal to switch to low-coverage in the presence of the premium subsidies.

**Result 2**: Premium subsidies can help induce any desirable separating equilibrium in the presence of asymmetric information by making the participation of producers with different attitudes towards risk in the policies designed for them incentive compatible.

The change in the structure of premium subsidies by ARPA can be viewed as an attempt to induce a different separating equilibrium. In 2000, the year before ARPA was enacted, the average per acre subsidy for low-coverage insurance (i.e., coverage below 80%) was $4.74, while the average subsidy for high-coverage insurance (i.e., coverage at or above 80%) was $2.49 per acre [7]. In 2001, the first year ARPA took effect, the average per acre subsidy for low-coverage insurance increased to $8.29 while the average per acre subsidy for high-coverage insurance increased to $9.23. The percent of acres in a high-coverage contract increased from 5.9% in 2000 to 9.3% in 2001, a 57.6% change [7].

### Premium subsidies as means of income redistribution

As Figs 2 and 3 show, the introduction of premium subsidies results in income transfers to producers that participate in crop insurance. These income transfers depend on the relative magnitude of the subsidies to the different crop insurance policies, *s*_*l*_*w*_*l*_ and *s*_*h*_*w*_*h*_, and are asymmetric across the different policy participants. Specifically, when *s*_*l*_*w*_*l*_ is less than *s*_*h*_*w*_*h*_ (case depicted in Fig 2), producers enrolling in crop insurance due to the subsidies gain $G_{si}^{l}=\int_{A_{l}^{'}}^{A_{l}}(ER_{ls}-ER_{si})dA$, producers with low coverage policy before and after the subsidies gain $G_{l}^{l}=\int_{Al}^{A_{h}^{'}}(ER_{ls}-ER_{l})dA$, producers switching from low to high coverage due to the subsidies gain $G_{l}^{h}=\int_{A_{h}^{'}}^{A_{h}}(ER_{hs}-ER_{l})dA$, and producers with high coverage before and after the subsidies gain $G_{h}^{h}=\int_{A_{h}}^{1}(ER_{hs}-ER_{h})dA$.

On the other hand, when *s*_*l*_*w*_*l*_ exceeds *s*_*h*_*w*_*h*_ (case depicted in Fig 3), producers enrolling in crop insurance due to the subsidies gain $G_{si}^{l}=\int_{A_{l}^{'}}^{A_{l}}(ER_{ls}-ER_{si})dA$, producers with low coverage policy before and after the subsidies gain $G_{l}^{l}=\int_{A_{l}}^{A_{h}}(ER_{ls}-ER_{l})dA$, producers switching from high to low coverage due to the subsidies gain $G_{h}^{l}=\int_{A_{h}}^{A_{h}^{'}}(ER_{ls}-ER_{h})dA$, and producers with high coverage before and after the subsidies gain $G_{h}^{h}=\int_{A_{h}^{'}}^{1}(ER_{hs}-ER_{h})dA$.

Since the welfare impacts of subsidies are asymmetric, the optimal *s*_*l*_ and *s*_*h*_ when the government seeks to transfer income to policy participants will be determined by the relative weight placed by the government on the different beneficiaries of the policy and the taxpayers who fund crop insurance [10, 31].

**Result 3**: Premium subsidies transfer income from taxpayers to agricultural producers that participate in crop insurance.

As noted earlier, premium subsidies have been growing over time and accounted for $6.26 billion and $6.36 billion in 2018 and 2019, respectively. The low-coverage insurance policies received $4.2 billion in 2018 and $4.32 billion in 2019, while high-coverage policies received $2.07 billion in 2018 and $2.04 billion in 2019 [7].

### Discussion of the stated and revealed roles of premium subsidies

As noted earlier, the stated role of premium subsidies is to increase producer participation in crop insurance. As our analysis has shown, however, if increased producer participation were the only goal of the government, it could have been achieved much more efficiently by providing subsidies to low-coverage insurance policies only (alternatively, removing the availability of high coverage contracts and, therefore, the subsidies associated with them, would result in the same outcome). Such a policy design would have still attracted the desired producer group to participate in crop insurance and would have avoided the income transfer to existing high-coverage insurance buyers. Based on Result 2, however, one could argue that the provision of subsidies to both low- and high-coverage insurance contracts is not evidence of a government desire to transfer income to more risk averse producers but that it reveals, instead, a desire to induce a certain separating equilibrium (that would not be attainable in the absence of *s*_*h*_).

Given that, as shown earlier (see Result 3), the introduction of the premium subsidies does result in income transfers from taxpayers to policy participants, the question that naturally arises (and is at the heart of this research) is whether these transfers are a goal or a necessity for the desired increased participation (and a separating equilibrium) to emerge. To answer this question, we need to evaluate whether the government could achieve the desired increased participation and any separating equilibrium at reduced costs. It turns out that it can, which makes income redistribution very much a goal of this government policy.

**Fig 4:**
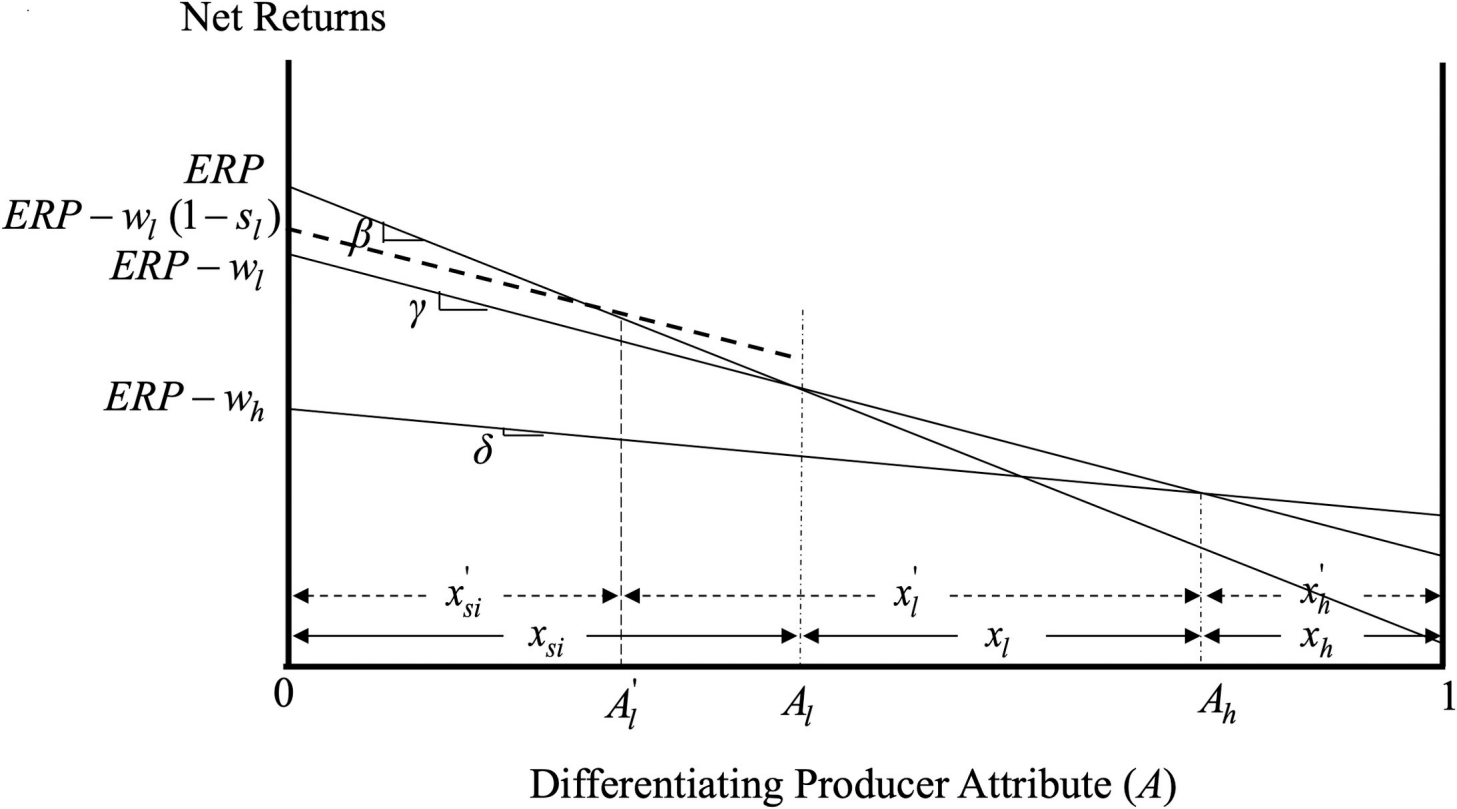
Mechanism that increases participation and maintains a separating equilibrium at reduced costs.

In particular, the government could have achieved the desired increase in producer participation by providing the premium subsidy *s*_*l*_ to new participants only. Without a subsidy paid to producers with low-coverage insurance already in the program, there would be no need for a subsidy for existing producers with high-coverage insurance to maintain the desired separating equilibrium (and the share/type of producers opting for high-coverage crop insurance). Fig 4 depicts this mechanism and shows how it can achieve an increased producer participation and maintain the desired separating equilibrium, while saving all the income transfers to existing policy participants identified in the previous section. It is important to note that, under this mechanism, the new policy participants would receive the premium subsidy for as long as the government desired their participation in crop insurance. Existing policy participants (who keep paying the same premium rate) would have no incentive to leave the program as their expected returns with crop insurance are greater than those without (see Fig 4). In addition, by participating in the program and purchasing a certain coverage level, a producer reveals their true type/level of risk aversion. If such producer were to leave the program one year, they would be able to reenter with the terms that were in place when they were participating (and would not be eligible for new subsidies designed to induce producers that used to self-insure to enter the program).

**Result 4:** Providing premium subsidies only to new policy participants can increase the efficiency of the policy in increasing producer participation in crop insurance and inducing a desired separating equilibrium.

At this point, it is important to reiterate that, while our analysis focuses on the introduction of new premium subsidies, our results are more general and apply also to cases where the government increases the magnitude of existing subsidies. In such a case, under our proposed mechanism, it is the increase in the premium subsidy associated with the low-coverage insurance that would be available only to new policy participants (while existing policy participants would keep paying the premium associated with their chosen insurance coverage policy, which includes the subsidies already in place). Put in a different way, in cases where the goverenment already subsidizes different insurance coverage policies, our proposed mechanism would not remove existing subsidies from current policy participants but would, instead, make the increase in the current premium subsidies available only to new policy participants.

To assess the magnitude of the savings associated with the implementation of our proposed policy design, we compare its costs to those of ARPA, focusing on the years before and after the implementation of this reform. RMA data indicates that ARPA increased producer acreage enrollment from 206.4 million acres in 2000 to 211.3 million acres in 2001 [7]. With the average premium subsidy for low-coverage insurance after ARPA being $8.29 per acre, our proposed policy design would require $40 million (4.9 million new acres times $8.29 per acre) of premium subsidies allocated to new participants for the same increase in acreage enrollment to be achieved. With ARPA costing 820 million (2001 premium subsidies of $1.77 billion less $951 million in 2000 premium subsidies), our policy design would have saved taxpayers $780 million, or 95% of the new subsidy payments in 2001 alone. Additional savings would be realized in subsequent years as premium subsidies under ARPA have continued to exist.

It is important to note that, in addition to being very efficient, this policy design is also very flexible. For instance, if an increase in the share of producers purchasing high-coverage policy were desired, it could be easily achieved by providing *s*_*h*_ to existing policy participants. Since *s*_*l*_ to existing policy participants would be zero under this mechanism, the amount of *s*_*h*_ that would be needed for the desired adjustment to occur would be smaller than that under the current policy design. The reason is that, as shown earlier, it is the difference between the subsidies to low- and high-coverage policies that determines the shares of producers opting for the different policies. Reducing one of them to zero (as the current participants are not subsidized under this mechanism) reduces also the size of the other subsidy that is required for the desired adjustment to occur.

The fact that the government could have achieved its objectives of increasing producer participation and inducing a desired separating equilibrium by subsidizing only new policy participants but chose to provide subsidies to all policy participants (both new and existing ones), reveals either that income redistribution through crop insurance is very much an objective of the government or that the current policy design constitutes a policy failure.

**Result 5:** Income transfers from taxpayers to agricultural producers are an objective of the crop insurance policy and not a necessary cost of increasing producer participation and inducing a desired separating equilibrium.

## Conclusions

This paper develops a model of heterogeneous producer attitudes towards risk to analyze different, stated and revealed, roles of crop insurance premium subsidies. The stated government objective of premium subsidies to increase producer participation in crop insurance is evaluated along with their role in inducing the desired producer behavior and a separating equilibrium in the presence of asymmetric information, and transferring income from taxpayers to agricultural producers/policy participants.

The analysis reveals a strong connection and a complementarity between the stated and revealed policy objectives of the government. Premium subsidies can, indeed, increase producer participation in the program, induce a (any) desired separating equilibrium with producers with different levels of risk aversion choosing different levels of risk coverage, and result in welfare transfers to agricultural producers.

The study then tests the assertion that the income transfers occurring through premium subsidies are a necessary cost for increasing producer participation in the program. To do so, we develop an alternative design of premium subsidies, one in which increased participation occurs through the provision of subsidies to new policy participants only. The proposed design can achieve the stated government objective of increased producer participation at reduced costs and rejects the idea that the income redistribution taking place under the current policy design is necessary for increasing producer participation in crop insurance.

In fact, the proposed alternative policy design can also be modified to induce any desired separating equilibrium, also at reduced costs. The presence of a policy design that can achieve increased producer participation and induce any desired separating equilibrium at reduced costs reveals that the premium subsidies in the current policy design are either a means of income redistribution or a policy failure.

## Supporting information

S1 Data Risk management agency data used in the analysis of the role of premium subsidies in crop insurance. (XLS)
